# Quantum interference in superposed lattices

**DOI:** 10.1073/pnas.2315787121

**Published:** 2024-02-05

**Authors:** Yejun Feng, Yishu Wang, T. F. Rosenbaum, P. B. Littlewood, Hua Chen

**Affiliations:** ^a^Okinawa Institute of Science and Technology Graduate University, Onna, Okinawa 904-0495, Japan; ^b^Department of Materials Science and Engineering, University of Tennessee, Knoxville, TN 37996; ^c^Department of Physics and Astronomy, University of Tennessee, Knoxville, TN 37996; ^d^Division of Physics, Mathematics, and Astronomy, California Institute of Technology, Pasadena, CA 91125; ^e^The James Franck Institute and Department of Physics, The University of Chicago, Chicago, IL 60637; ^f^School of Physics and Astronomy, University of St Andrews, St Andrews KY16 9SS, United Kingdom; ^g^Department of Physics, Colorado State University, Fort Collins, CO 80523

**Keywords:** π-phase shift, quantum oscillations, incommensurate reciprocal lattices, fermiology

## Abstract

Charge transport in solids at low temperature reveals a material’s mesoscopic properties and structure. Under a magnetic field, Shubnikov–de Haas (SdH) oscillations inform complex quantum transport phenomena that are not limited by the ground state characteristics and have facilitated extensive explorations of quantum and topological interest in two- and three-dimensional materials. Here, in elemental metal Cr with two incommensurately superposed lattices of ions and a spin-density-wave ground state, we reveal that the phases of several low-frequency SdH oscillations in $\sigma_{\mathrm{xx}}\;\left(\rho_{\mathrm{xx}}\right)$ and $\sigma_{\mathrm{yy}}\;\left(\rho_{\mathrm{yy}}\right)$ are no longer identical but opposite. These relationships contrast with the SdH oscillations from normal cyclotron orbits that maintain identical phases between $\sigma_{\mathrm{xx}}\;\left(\rho_{\mathrm{xx}}\right)$ and $\sigma_{\mathrm{yy}}\;\left(\rho_{\mathrm{yy}}\right)$ . We trace the origin of the low-frequency SdH oscillations to quantum interference effects arising from the incommensurate orbits of Cr’s superposed reciprocal lattices and explain the observed π-phase shift by the reconnection of anisotropic joint open and closed orbits.

## Significance

Understanding the Fermi surface is of fundamental importance to metals. Here, we explore a situation where two sets of incommensurate lattices are superposed together in the elemental metal Cr. These two reciprocal lattices are connected through many-body electron correlations that have distinct experimental signatures. They combine to build a Fermi surface with many small de Haas–van Alphen orbits and quantum interference paths that are not detectable by standard photoemission techniques. In the spin- and charge-density-wave state of Cr, this construction leads to Shubnikov–de Haas oscillations with opposite phases between two orthogonal channels of transverse magnetoresistance. This phenomenon represents a natural three-dimensional analogue to topological and Moiré systems.

Functional materials with interesting and useful electronic, magnetic, and optical responses can be created through the engineering of their electronic structure (1–3). While conventional crystalline materials still hold many hidden degrees of freedom for unconventional quasiparticles of topological nature (1), an alternative route to access complex electronic behavior is to artificially superpose lattices upon each other to avoid the confinement of the three-dimensional space group symmetry (2, 3). Examples include the control of the termination layer at the molecular-beam-epitaxy growth interface of two insulating oxides (2) and the Moiré construction of two graphene sheets twisted at small angles (3). New properties peculiar to the composite lattice can emerge, such as superconductivity (4, 5).

These examples suggest the power of exploring degrees of freedom beyond the examples cited above to construct unique types of composite lattices. One natural but largely unexplored example exists in incommensurately modulated crystalline materials (6). For conventional crystals, the electronic structures are dictated by their three-dimensional space groups (1). The symmetry property of an incommensurate structure, on the other hand, is mathematically constructed from space groups of a higher dimensional space, before being sectioned into three dimensions (7). Many incommensurate structures exist in metals, such as helical magnets and spin and charge density waves (6, 8, 9). The incommensurate superstructure involving either charge or spin introduces a second set of reciprocal lattices which interacts with the underlying ions. While historically treated as simply opening a gap at the Femi surface of the first set of reciprocal lattices (10–12), multiple applications of the superposed incommensurate reciprocal lattice vectors can provide for more complex possibilities and properties. As we detail below, sophisticated galvanomagnetic behavior emerges from such composite electronic structures because of the incommensurate nature of their superposition.

**Fig. 1.**
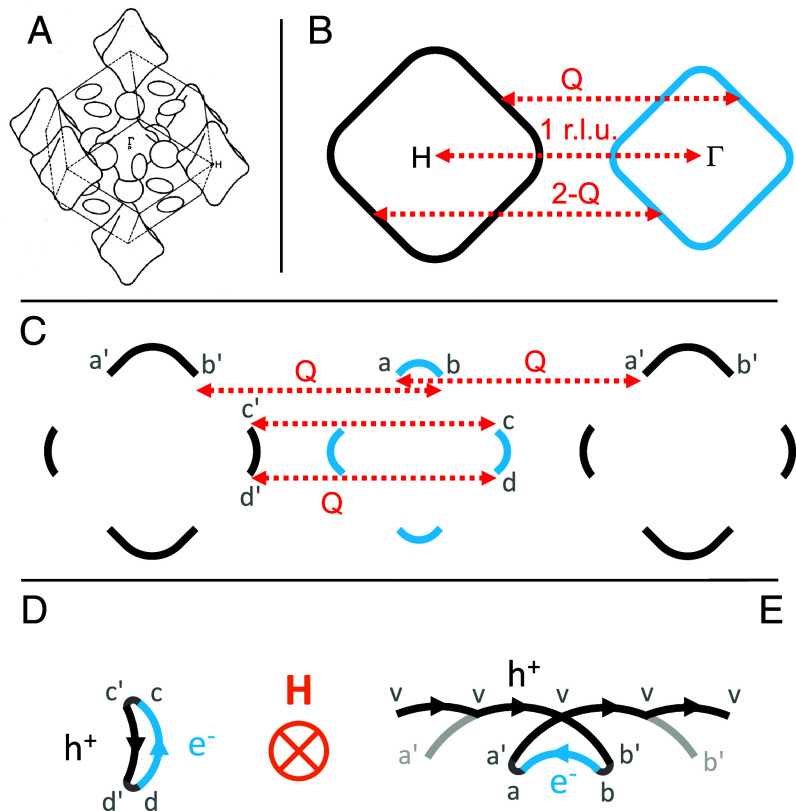
Fermi surface and cyclotron orbits of two superposed reciprocal lattices. (*A*) Calculated paramagnetic band structure of Cr, which is composed of closed octahedral and ellipsoidal forms of hole and electron pockets, is similar to those of Mo and W. The schematic is adapted from ref. 13. (*B*) A schematic of octahedral Fermi surfaces of hole (black) and electron (blue) projected onto the *a-b* plane. Their parallel surfaces are matched through the nesting wave vector **Q**. (*C*) Upon the formation of a SDW state, the long-range antiferromagnetic order introduces a second set of reciprocal lattices, with the wave vector **Q** serving as the basis. **Q** connects fragmented electron and hole Fermi surfaces in the original ionic reciprocal lattice into continuous forms in panels (*D*) and (*E*). (*D*) Under field, carriers flow along the “banana” orbit “*c-c′-d′-d-c*,” as tips of the electron and hole octahedra are not annihilated by the nesting condition. (*E*) Another “triangle” orbit is formed when two pieces of large hole arc (*a’-b’*) and a small electron arc (*a-b*) are connected into a closed loop “*b-a-a′-v-b′-b.*” Here two hole-arcs “*a′-b′*” are relatively displaced by 2**Q**, and a gap opens at the band crossing point *v*. A full superposition of the two reciprocal lattices would create an open orbit “…*v-v-v-v-v*….”

We explore unconventional electronic characteristics in the archetypical spin-density-wave (SDW) system, Cr. Cr possesses a simple body-centered cubic Bravais lattice and a one-atom basis, which allows a high-fidelity theoretical understanding of its paramagnetic band structure. The paramagnetic Fermi surface is composed of only closed forms with no open sheets and is isomorphic to those of W and Mo (Fig. 1*A*) (8, 13). Below *T*_N_ = 311.5 K, long-range SDW order with an incommensurate wavevector **Q** = (0.952, 0, 0) develops in Cr as the result of a nesting instability at the Fermi surface (Fig. 1) (8, 12). Because of different sizes of the hole and electron octahedra, the paramagnetic Fermi surface is imperfectly gapped with residual fragments of both carrier types (Fig. 1).

We find that the superposed reciprocal lattices of the ionic lattice and the SDW lead to two low-frequency SdH oscillations of 36 T and 40 T, with different galvanomagnetic behavior in field and temperature dependences, anisotropy, and existence of harmonics. Despite their differences, the SdH oscillations at each frequency reliably demonstrate opposite phases (or equivalently, a π-phase shift) between two configurations of electrical current **I** flowing either parallel or perpendicular to **Q**. In each case, the external magnetic field **H** is along a cubic axis and perpendicular to both **I** and **Q**. For both the SdH and dHvA oscillations, their tiny areas, in the range of 260 to 440 ppm of the first Brillouin zone cross-section, represent a construction through multiple applications of wavevector **Q** over the fragmented original Fermi surface, a mechanism generic for metals with incommensurate superlattices. We attribute these sub-50 T SdH oscillations to a quantum interference effect in a fractal network of orbits. The opposite phase condition between the different resistivity channels is explained by further considerations of anisotropic conduction in joint open and closed orbits in the electronic structure, moving beyond the simple application of either conventional or Berry-phase theories. This quantum transport phenomenon should be able to be engineered more generally in appropriately configured quantum and topological materials.

## Results

### Single Q-Domain of SDW Cr.

**Fig. 2.**
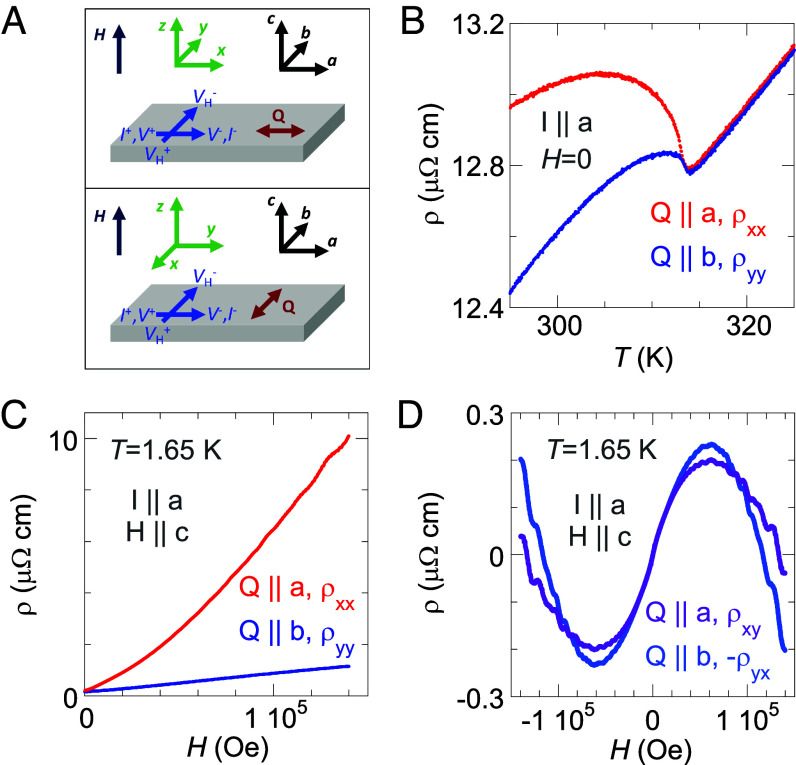
Full resistivity matrix ρ measured through single Q-domain control. (*A*) A bar-shaped single-crystal Cr sample (gray), with the cubic lattice structure marked by the coordinates “*a-b-c*” (black), is wired under a six-lead configuration for both Hall and MR measurements (blue marking); the real samples are listed in *SI Appendix*, Fig. S1. Separate single Q-domains (red) along two different cubic axes (*Materials and Methods*) permit us to measure the full resistivity matrix ρ in the H‖z geometry with a fixed lead placement. Coordinates *x-y-z* (green) of ρ are always defined by orientations of **Q** and **H** as Q‖x and H‖z . (*B*) Zero-field resistivities of Cr in a single Q-state are contrasted near *T*_N_ between Q‖I (red) and Q⊥I (blue) configurations and can be compared to the literature (16). (*C*) Magnetoresistivities $\rho_{\mathrm{xx}}\left(H_{z}\right)$ and $\rho_{\mathrm{yy}}\left(H_{z}\right)$ reveal a large anisotropy, yet both are much larger than (*D*) the Hall resistivities $\rho_{\mathrm{xy}}\left(H_{z}\right)$ and ${-\rho }_{\mathrm{yx}}$ . The consistency between Hall resistivities $\rho_{\mathrm{xy}}$ and ${-\rho }_{\mathrm{yx}}$ verifies our control of a single Q-state as the measurements were performed with **Q** along two different axes with a constant lead configuration. The specific **I**-**Q**-**H** configurations for measured ρ matrix elements are individually listed in the panels.

Despite the threefold degeneracy of **Q** under the cubic symmetry, a proper and verifiable field-cooling procedure reliably induces a single Q-domain along one of the cubic axes, with a volume ratio greater than 98% [(14–16), *Materials and Methods*]. The state of a single Q-domain allows measurement conditions to be specified uniquely by the configuration of **Q**, electrical current **I**, and magnetic field **H** (Fig. 2). For a single crystal specimen wired in a standard six-lead configuration for both MR and Hall measurements (Fig. 2, *Materials and Methods*, *SI Appendix*, Fig. S1), switching the single Q-domain between different cubic axes provides a technical advantage to fully access the resistivity matrix $\rho \left(H_{z}\right)=\left[\begin{array}{cc} \rho_{\mathrm{xx}} & \rho_{\mathrm{xy}}\\ \rho_{\mathrm{yx}} & \rho_{\mathrm{yy}} \end{array}\right]$   in a coordinate system defined by Q ‖x   , and H ‖z   , as the fixed lead placement largely avoids systematic errors. We follow this convention of *x-y-z* from here on. Hall resistivities $\rho_{\mathrm{xy}}$   and $-\rho_{\mathrm{yx}}$   , measured under Q ‖I   and Q ⊥I   configurations, respectively, provide the experimental verification of our single-Q procedure by confirming the current-voltage reciprocity $\rho_{\mathrm{xy}}=-\rho_{\mathrm{yx}}$   (Fig. 2). Our measured $\rho_{\mathrm{xy}}$   and $-\rho_{\mathrm{yx}}$   are consistent with results reported in the literature under the identical **H**-**I**-**Q** configuration (14, 17), and the nonmonotonic field dependence indicates carriers of different mobilities transitioning from limits of low to high field.

As the **Q** wavevector breaks the fourfold symmetry in the *x-y* plane, a large anisotropy exists between $\rho_{\mathrm{xx}}$ and $\rho_{\mathrm{yy}}$ even at zero field (16). The contrast in our measured resistivities $\rho_{\mathrm{xx}}$ and $\rho_{\mathrm{yy}}$ (Fig. 2*B*) is more pronounced than that reported in the literature (16), testifying to the completeness of our single Q-domain state. Under field at *T* = 1.65 K, the anisotropy remains between $\rho_{\mathrm{xx}}\left(H_{z}\right)$ and $\rho_{\mathrm{yy}}\left(H_{z}\right)$ (Fig. 2*C*), which is consistent with what is reported in the literature between Q‖I and Q⊥I (14).

### π-Phase Shifts between SdH Oscillations.

**Fig. 3.**
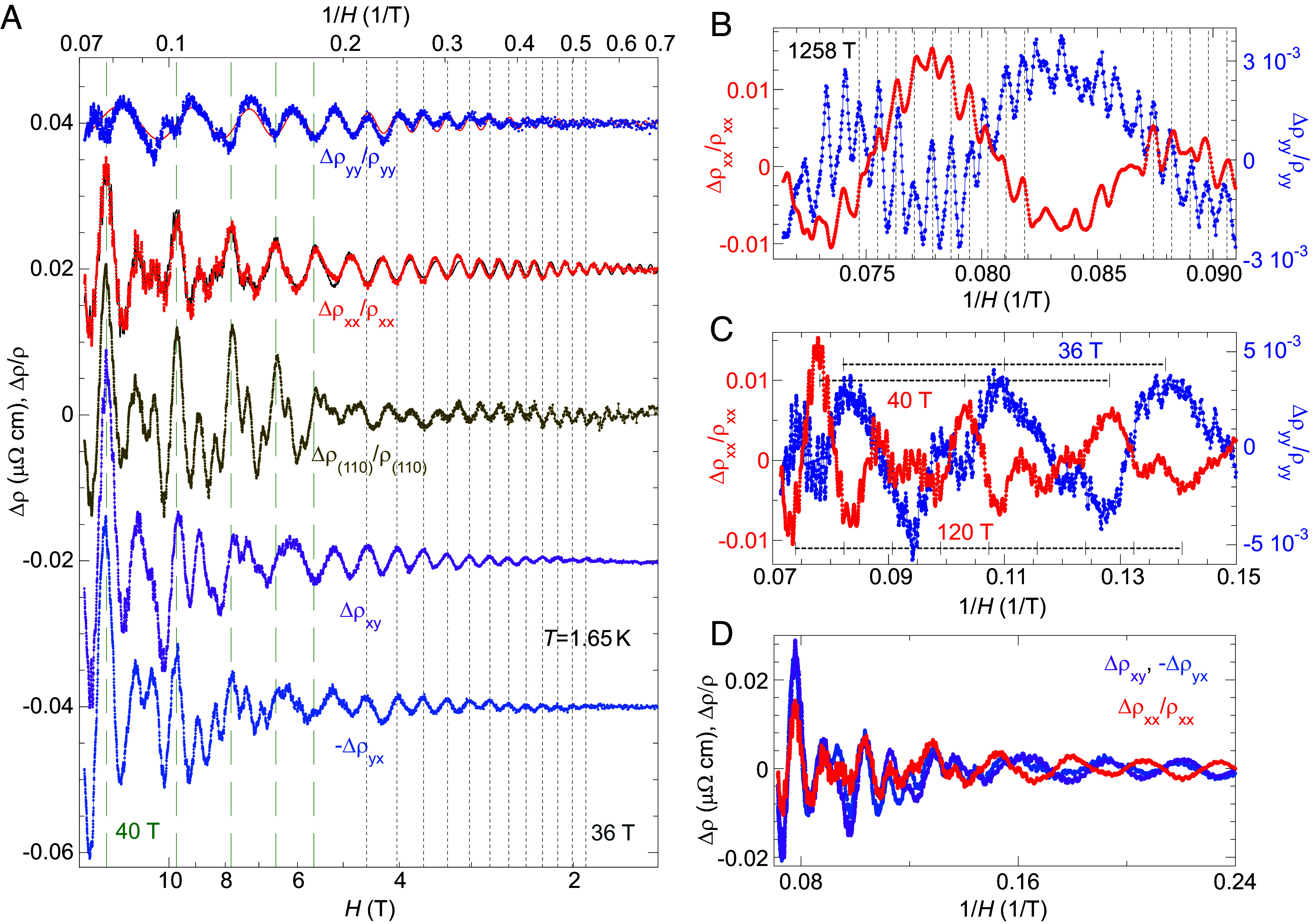
π-phase shifts between SdH oscillations of $\rho_{\mathrm{xx}}$   and $\rho_{\mathrm{yy}}$. (*A*) Raw SdH oscillations of $\Delta \rho_{\mathrm{xx}}/\rho_{\mathrm{xx}}$, $\Delta \rho_{\mathrm{yy}}/\rho_{\mathrm{yy}}$, $\Delta \rho_{\left(110\right)}/\rho_{\left(110\right)}$, $\Delta \rho_{\mathrm{xy}}$, and $-\Delta \rho_{\mathrm{yx}}$, vertically displaced for clarity, are plotted vs. *H*, with corresponding 1/*H* values marked on the top. A log-*x* scale is used to demonstrate the high field behavior. Two sets of vertical lines mark the periodicities of 36 T (gray short dash) and 40 T (green long dash) oscillations which are prominent in different regions of field. For *H* < 6 T, all SdH oscillations are dominated by the 36 T mode. The 40 T frequency is very prominent with $\Delta \rho_{\left(110\right)}/\rho_{\left(110\right)}$ , together with the 120 T oscillations in between. $\Delta \rho_{\mathrm{yy}}/\rho_{\mathrm{yy}}$ is fit with a single frequency of 36 T with an exponentially decaying amplitude (red line) to demonstrate its dominant presence in that channel. $\Delta \rho_{\mathrm{xx}}/\rho_{\mathrm{xx}}$ is fit with six frequencies (*Materials and Methods*) to demonstrate the extraction of phases of the SdH oscillations. The phase of the 36 T oscillation in $\rho_{\mathrm{yy}}$ is opposite to those of $\rho_{\mathrm{xx}}$ and $\rho_{\left(110\right)}$ , but identical with those of $\rho_{\mathrm{xy}}$ and ${-\rho }_{\mathrm{yx}}$ . (*B*) For a normal orbit of 1,258 T, SdH oscillations in $\rho_{\mathrm{xx}}$ and $\rho_{\mathrm{yy}}$ are in phase, as indicated by the set of vertical dashed lines that are separated by the oscillation period. (*C*) Periods of 36 T, 40 T, and 120 T oscillations are marked for $\Delta \rho_{\mathrm{xx}}/\rho_{\mathrm{xx}}$ and $\Delta \rho_{\mathrm{yy}}/\rho_{\mathrm{yy}}$ in the high field range. With ticks on the scale bar marking the local minima and maxima positions of $\rho_{\mathrm{xx}}$ and $\rho_{\mathrm{yy}},$ respectively, the 120 T SdH oscillations can be verified visually to be of opposite phase (a π-phase shift). (*D*) Between $\rho_{\mathrm{xx}}$ and $\rho_{\mathrm{xy}}$ ( ${-\rho }_{\mathrm{yx}})$ , both the 40 and 120 T SdH oscillations are visibly in phase at high field, but 36 T oscillations between these two channels have a π-phase shift that becomes clear at low field.

**Fig. 4.**
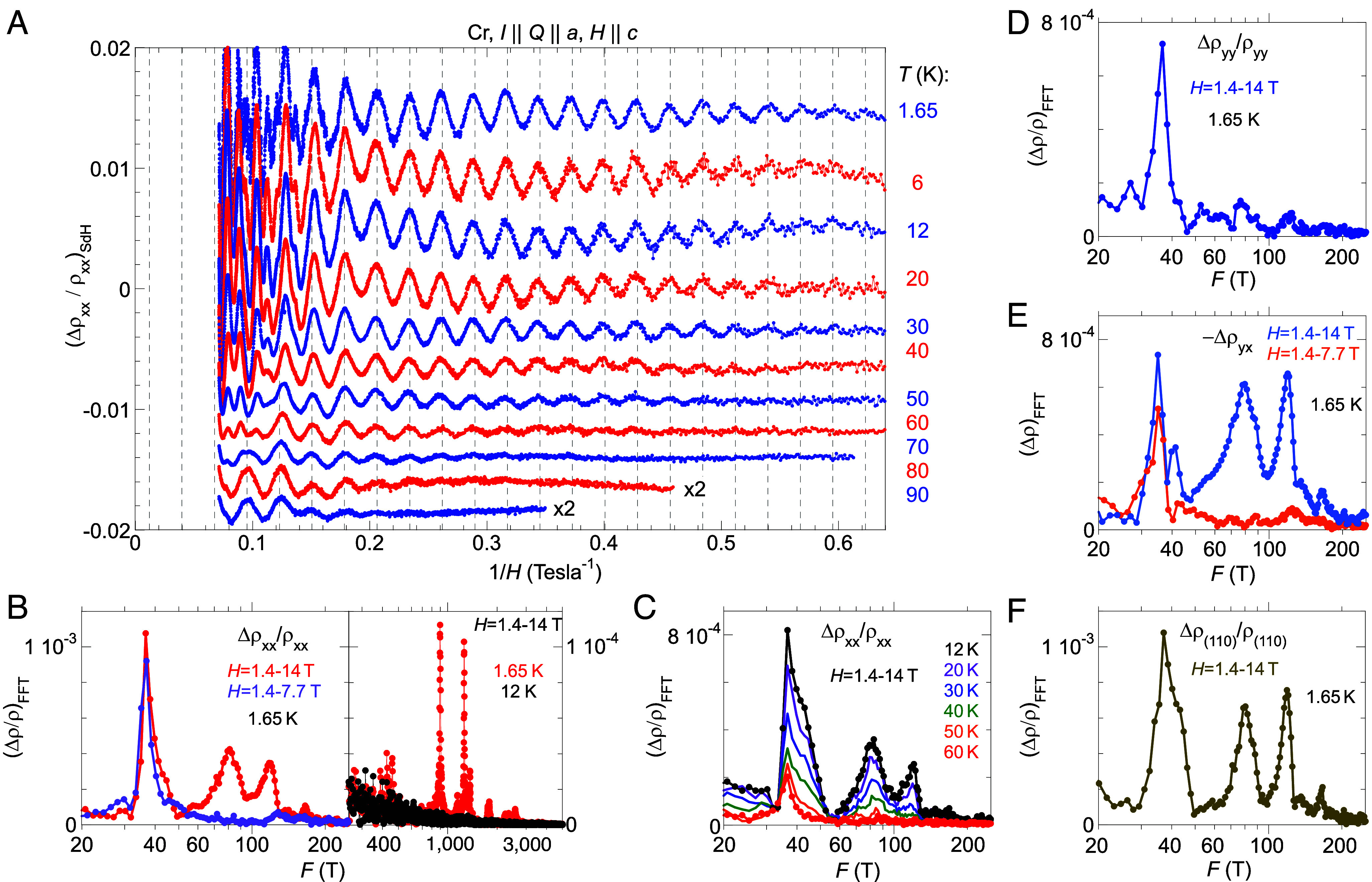
Low-frequency SdH oscillations. (*A*) The temperature evolution of $\Delta \rho_{\mathrm{xx}}/\rho_{\mathrm{xx}}$ is plotted vs. 1/*H* from *T* = 1.65 to 90 K. All data are separated vertically for clarity. The vertical dashed lines mark the maximal position of $\Delta \rho_{\mathrm{xx}}/\rho_{\mathrm{xx}}$ in a regular spacing of 1/36.0 T^-1^. (*B*) FFT spectra of $\Delta \rho_{\mathrm{xx}}/\rho_{\mathrm{xx}}$ reveal two SdH frequencies of 36 T and 40 T, and higher harmonics of the latter at 80 T and 120 T. These low-frequency SdH oscillations (left-hand *y*-scale) dominate higher-frequency quantum oscillations in $\Delta \rho_{\mathrm{xx}}/\rho_{\mathrm{xx}}$ (right-hand *y*-scale) by a factor ~10. The 40 T oscillation and its two harmonics exist only in the high field range above 7.7 T, while the 36 T oscillation exists down to ~1.5 T. (*C*) The 36 T and 40 T SdH oscillations exist to high temperature of 90 K and 60 K, respectively; their temperature evolution provides a clean separation. The harmonics 80/120 T share the same temperature evolution as the 40 T oscillation. (*D*–*F*) FFT spectra of $\Delta \rho_{\mathrm{yy}}/\rho_{\mathrm{yy}}$ , $-\Delta \rho_{\mathrm{yx}}$ , and $\Delta \rho_{\left(110\right)}/\rho_{\left(110\right)}$ display characteristics of the 36 T and 40 T oscillations. $\rho_{\left(110\right)}\left(H_{z}\right)$ is the MR with current **I** flowing parallel to the (1,1,0) axis, and was measured on a separate piece of Cr single crystal (*SI Appendix*, Fig. S1*B*). It demonstrates that the SdH frequencies of 36 T and 40 T, and harmonics 80 T and 120 T, are repeatable and sample independent.

From all four matrix elements of $\rho \left(H_{z}\right)$   at 1.65 K, SdH oscillations are extracted and plotted vs. 1/*H* in Fig. 3. For many SdH frequencies, the phase relationship in $\rho_{\mathrm{xx}}$   , $\rho_{\mathrm{yy}}$   , and $\rho_{\mathrm{xy}}$   ( $-\rho_{\mathrm{yx}}$   ) can be directly visualized and compared. There are two types of behavior. For high frequency (1,258 T), the phases of SdH oscillations in $\rho_{\mathrm{xx}}$   and $\rho_{\mathrm{yy}}$   are identical (Fig. 3*B*). However, for low-frequency (36 T) SdH oscillations, there exists an unusual opposite phase relationship (or a π  -phase shift) between $\rho_{\mathrm{xx}}$   and $\rho_{\mathrm{yy}}$   , which can be seen directly by inspecting the raw data in Fig. 3*A*. Through fast Fourier transform (FFT) analysis (Fig. 4) over different field ranges, measurement geometries, and temperatures, we find that there exist both 36 T and a set of 40/80/120 T frequencies in each element of ρ   , with 80 T and 120 T being the harmonics of 40 T. There exists one SdH study in the literature (18), which likely suggests the existence of a 40 T mode in $\rho_{\mathrm{xx}}$   with a 120 T mode as its third harmonic. However, it did not resolve multiple frequencies at sub-100 T. Here our analysis presents a detailed picture of SdH oscillations at low frequency. While the opposite phase relationship of the 120 T oscillation is clearly observable in the raw data (Fig. 3*C*), it is difficult to directly visualize the 40 and 80 T oscillations in $\rho_{\mathrm{yy}}$   . Here we extract precise phases of several of the strongest SdH oscillations through direct fitting of the raw SdH data (*Materials and Methods*, *SI Appendix*, Fig. S3). Overall, all four frequencies of 36/40/80/120 T have a π-phase shift between $\rho_{\mathrm{xx}}$ and $\rho_{\mathrm{yy}}$ , while the phases at 1,258 T are identical (*SI Appendix*, Fig. S3). For $\rho_{\mathrm{xy}}$ ( $-\rho_{\mathrm{yx}}$ ), it is visually verifiable that the phase of the 36 T oscillations matches that of $\rho_{\mathrm{yy}},$ while the phase of the 120 T oscillations is identical to that of $\rho_{\mathrm{xx}}$ (Fig. 3*D*).

Our discussion of SdH oscillations has to this point focused on the resistivity matrix ρ , instead of the conductivity matrix σ (19). A full calculation of σ verifies that the π-phase shifts between SdH oscillations in $\rho_{\mathrm{xx}}$ and $\rho_{\mathrm{yy}}$ also exist between $\sigma_{\mathrm{xx}}$ and $\sigma_{\mathrm{yy}}$ (*SI Appendix*, Fig. S2). Indeed, we have a relatively simple situation in three-dimensional Cr; both $\rho_{\mathrm{yy}}$ and $\rho_{\mathrm{xx}}$ in Cr dominate over $\rho_{\mathrm{xy}}$ (Fig. 2). As $|\rho_{\mathrm{xy}}\rho_{\mathrm{yx}}/\rho_{\mathrm{xx}}\rho_{\mathrm{yy}}|<6\%$ at all fields, we have $\sigma_{\mathrm{xx}}\;∼1/\rho_{\mathrm{xx}}$ and ${\sigma_{\mathrm{yy}}\;∼1/\rho }_{\mathrm{yy}}$ (19). Moreover, given the conventional phase relationship of the 1,258 T SdH oscillations between $\rho_{\mathrm{xx}}$ and $\rho_{\mathrm{yy}}$ (Fig. 3*B*), the opposite phases of the 36/40/80/120 T oscillations between *x* and *y* channels cannot be attributed to the matrix inversion. Instead, the data reveal the genuine characteristics of an unexpected transport phenomenon.

We have made additional checks by measuring the magnetoresistivity $\rho_{\left(110\right)}$   from a different piece of a Cr single crystal of a different origin, with **I** || (1,1,0), **Q** || (1,0,0), and **H** || (0,0,1) (Fig. 3, *Materials and Methods*, *SI Appendix*, Fig. S1*B*). Both the 36 T and 40 T SdH oscillations are confirmed in $\rho_{\left(110\right)}$ , together with the higher harmonics at 80 T and 120 T, and other ordinary frequencies (Figs. 3 and 4 and *SI Appendix*, Table S1). From the raw SdH oscillations (Fig. 3), the 36.0 T frequency can be verified to a precision within ±0.2 T. The observed SdH oscillations reveal that the phases of the 36, 40, 80, and 120 T orbits remain identical to those of $\rho_{\mathrm{xx}}$ (Fig. 3). Hence, the phases of all these low-frequency oscillations should follow one of the binary values in $\rho_{\mathrm{xx}}$ and $\rho_{\mathrm{yy}}$ , rather than continuously varying over π with different current **I** directions. The MR measured along all other directions in the *x-y* plane can be considered as a linear combination of $\rho_{\mathrm{xx}}$ and $\rho_{\mathrm{yy}}$ . We return to this topic in *Discussion*.

We show the temperature evolution of ${\Delta \rho }_{\mathrm{xx}}\left(H_{z}\right)/\rho_{\mathrm{xx}}$ in Fig. 4. The four low-frequency oscillations dominate the SdH response, as at *T* = 1.65 K. The Δρ/ρ amplitudes of all high-frequency SdH oscillations (> 150 T) in $\rho_{\mathrm{xx}}$ are weaker than that of the 36T mode by at least a factor of 10 (Fig. 4*B*). All other SdH oscillations disappear below our sensitivity level at ~12 K (Fig. 4*B*), while the 40 T family of oscillations disappears at ~70 K in $\rho_{\mathrm{xx}}$ (Fig. 4 *A* and *C*), and the 36 T oscillation remains noticeable at 90 K (Fig. 4*A*).

Despite being close in frequency, the 36 T and 40 T SdH oscillations have very different characteristics, which allows an experimental separation of the two. At 1.65 K, the 36 T mode has comparable amplitude ratios between ${\Delta \rho }_{\mathrm{xx}}/\rho_{\mathrm{xx}}$ (1.1 × 10^−3^ in Fig. 4*B*) and ${\Delta \rho }_{\mathrm{yy}}/\rho_{\mathrm{yy}}$ (8 × 10^−4^ in Fig. 4*D*). It is noticeable from 1.5 T up with a mild field dependence and no detectable higher harmonics (Figs. 3 and 4). By comparison, the 40 T oscillation is always accompanied by strong harmonics at 80 T and 120 T and all three are only observable above ~7 T in our samples (Figs. 3 and 4). The 40 T oscillation and its harmonics have strong amplitudes in both $\rho_{\mathrm{xx}}$ and $\rho_{\mathrm{xy}}$ ( $-\rho_{\mathrm{yx}}$ ) but are weak in $\rho_{\mathrm{yy}}$.

### dHvA Oscillations.

**Fig. 5.**
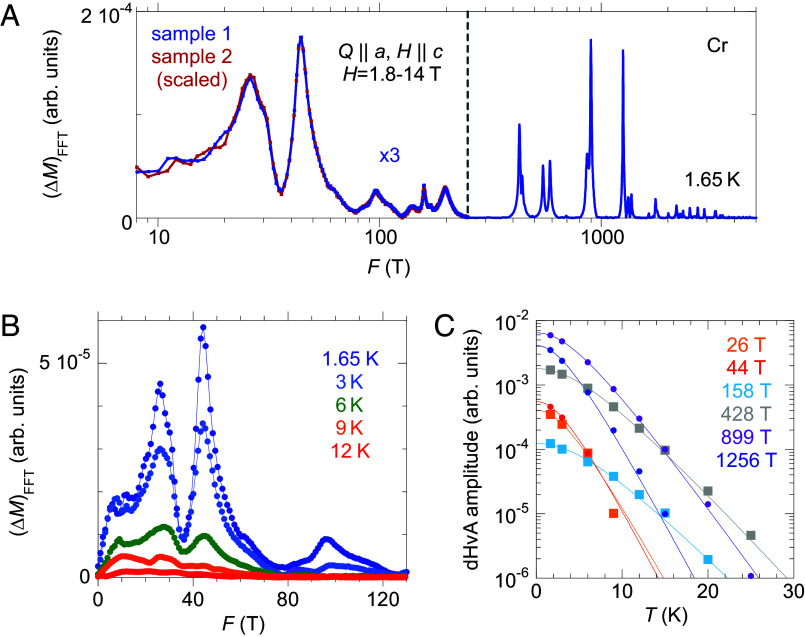
dHvA oscillations. (*A*) The full dHvA spectrum under the specified field-Q configuration. Data from two samples (*Materials and Methods*, *SI Appendix*, Figs. S1 *C* and *D*) are consistent, revealing low-frequency modes of 26, 32, and 44 T, in addition to other high frequency modes. *SI Appendix*, Table S1 provides a comparison of dHvA frequencies in the literature and our measured dHvA and SdH frequencies. Amplitudes of low-frequency dHvA oscillations are plotted with a scaling factor of 3 for sample 1. The dHvA amplitude of sample 2 is scaled to that of sample 1. (*B* and *C*) Temperature dependence of dHvA oscillations of panel (*A*). For panel (*C*), intensities of 26 T and 32 T are integrated together. Side peaks of 428 T and 899 T at 440 T and 862 T are also integrated with the main frequency, respectively, as they cannot be differentiated at higher temperatures.

The paramagnetic Fermi surface of Cr is isomorphic to those of Mo and W, as all are composed of closed forms and differ in energy only by the varying strength of spin–orbit coupling (20–22). Under the same configuration of **H** || (0,0,1), both Mo and W have multiple dHvA frequencies in the range of 10,000 to 25,000 T from the large hole and electron octahedra, but no dHvA frequency below 500 T (20–22). For Cr in the single Q-state under the Q⊥H configuration, there is no dHvA quantum oscillation frequency higher than 4,000 T (Fig. 5*A*). Instead, refs. 15 and 23 reported that two frequencies under 50 T emerge (*SI Appendix*, Table S1), indicating that the large octahedral Fermi surfaces are partially gapped in the SDW state and the sub-50 T frequencies are created by the **Q** vector.

Our precise control of the single Q-domain state provides the opportunity to measure dHvA oscillations in comparison with their SdH counterparts. We have detected dHvA oscillations on two Cr samples (Fig. 5*A*, *Materials and Methods*, *SI Appendix*, Fig. S1), and the frequencies above 150 T are consistent with reported values in the literature (15, 23) (*SI Appendix*, Table S1). Most importantly, we reveal several low-frequency dHvA oscillations at 26, 32, and 44 T (Fig. 5). Our measured SdH and dHvA frequencies are consistent within each technique, over multiple samples and multiple cooldowns of each. The differences between sub-50 T quantum oscillations observed by dHvA and SdH techniques are larger than the experimental uncertainty and represent a real discrepancy.

A study of the temperature dependence of the dHvA oscillations reveals further differences to the SdH results (Fig. 5*C*). For six major orbits that represent both the original paramagnetic Fermi surface (428, 899, and 1,256 T) and the **Q**-reconstructed Fermi surface (26, 44, and 158 T), all amplitudes reduce with temperature in a similar fashion, indicating similar and finite cyclotron masses. All sub-100 T modes are no longer detectable at ~10 K, while only the 428 T and 899 T dHvA oscillations are noticeable at 25 K (Fig. 5). This behavior sharply contrasts with that of the SdH modes as they disappear above 90 K and 60 K, respectively (Fig. 3). Our measurements suggest that the SdH oscillations of 36 T and 40 T (Fig. 3) are unlikely to share the same orbits of the dHvA oscillations (Fig. 5).

## Discussion

### Q-Induced Composite dHvA/SdH Orbits.

No low-frequency SdH/dHvA oscillations are expected to arise from the paramagnetic Fermi surface. Hence, we first discuss generic schemes of how such orbits can be constructed through the wave vector **Q**, using the residual fragments of the Fermi surface that remain after electron and hole octahedra have been imperfectly gapped by the SDW (15, 23, 24). Historically it has been assumed that either the electron octahedral surface was fully destroyed by the incommensurate SDW or, alternatively, a **Q**-induced band structure reconstruction was applied to hole ellipsoids that do not relate to the SDW gap, leading to much larger suggested SdH/dHvA frequencies of 100 to 200 T. Our measured SdH/dHvA periods between 26 and 44 T represent a cyclotron cross-section *S* of 2.5 to 4.2 × 10^−3^ Å^−2^, which is about 260 to 440 ppm of the first Brillouin zone cross-section area ( $8\pi^{2}/a^{2}$   with the *bcc* lattice constant *a* = 2.882 Å). Connected by only the primary **Q** vector, a potential orbit can be formed through residual fragments of the nested octahedra along the cubic axis (1, 0, 0) (Fig. 1*D*), which has been referred to as the “banana” orbit (10, 11). As the lateral sizes of dHvA/SdH orbits are about 0.02 r.l.u., while ( 1-Q)≅   0.05 r.l.u., other types of orbits can be constructed through a combination of shiftings by multiples of **Q**. For example, an orbit shaped as a “triangle” is formed by an electron segment in addition to two hole-segments displaced relatively by 2**Q**, opening a gap at point *v* to avoid band crossing (Fig. 1*E*). On these orbits, charge carriers travel alternately from electron to hole segments, circling a closed loop connected by the primary path and multiples of **Q**, thereby creating low-frequency dHvA oscillations.

Schemes of Fermi surface reconstruction are complicated by the unknown sizes of Fermi surface fragments after the SDW gap is created. Unfortunately, an existing photoemission study on Cr films (25) did not clearly resolve the gapped area and did not have a sufficiently fine resolution in reciprocal space to define the Fermi surface fragment to the degree needed here. Comparing to dHvA oscillations in the 90 to 250 T range, which were previously regarded as rising from **Q**-reconstruction of hole ellipsoids (14, 15, 24), the dHvA oscillations of 26 to 44 T have surprisingly large amplitudes (Fig. 5*A*). As none of the cyclotron masses are particularly small, these large dHvA amplitudes of closely spaced frequencies imply a large Landau level DOS from many degenerate orbits in the reconstructed electronic structure (equations 2.68 and 2.69 of ref. 26).

We further note that the sub-50 T SdH oscillations are different in frequency from the dHvA oscillations and survive to the higher temperatures of 60 to 90 K as compared to the 12 K of the dHvA oscillations. Taken together, we deduce that instead of a conventional interpretation of extremely light carriers, these SdH features are likely created by quantum interference effects (26–28). The quantum interference introduces SdH oscillations that do not have dHvA correspondences and labels carriers with seemingly low masses (26). Quantum interference was previously suggested for Cr (24). However, the raw SdH oscillations of $\rho_{\mathrm{yy}}$ in ref. 24 have overly large amplitudes for a configuration with an open orbit. In addition, ref. 24 only focused on SdH frequencies above 100 T and identified the 120 T frequency as a primary orbit with a specified quantum interference area (despite ref. 18’s suggestion that it was the third harmonic of 40 T). Our study indicates that in $\rho_{\mathrm{xx}}$ , $\rho_{\mathrm{yy}}$ , and $\rho_{\left(110\right)}$ , only the 36 T and 40 T SdH oscillations are due to a quantum interference effect.

Because the Q-vector is incommensurate with the paramagnetic unit cell, the existence of “orbits” as shown in Fig. 1 is a simplification. In the absence of scattering, a quasiparticle on the Fermi surface will peregrinate in an aperiodic fashion and orbits will never close. Effective closed orbits (such as Fig. 1*D*) and periodic orbits (such as Fig. 1*E*) result from an approximation where only the lowest-order coupling of a quasiparticle to the SDW is taken into account. Because SdH is a resistive (scattering) measurement, the effective orbits observed will be weighted by the mean free time before momentum-nonconserving (impurity, phonon) scattering resets the quasiparticle momentum into the rest frame. In contrast, dHvA is a thermodynamic property and is weighted by the density of states; impurity scattering will broaden what is in principle a fractal-like forest of peaks and the dominant frequencies will not be identical to those seen in SdH. It is the reconnection of orbits via scattering or tunneling processes loosely labeled “magnetic breakdown” (29), that allows periodic behavior to emerge. As we now show, the important influence of reconnections is manifested in the strong anisotropy and the π-phase shift.

### An Anisotropic Origin of the π-Phase Shift.

Both dHvA and quantum interference oscillations represent quantization by a nonlocal quantity of loop size (26, 27). Therefore, SdH oscillations from two orthogonal channels, $\rho_{\mathrm{xx}}$ ( $\sigma_{\mathrm{xx}}$ ) and $\rho_{\mathrm{yy}}$ ( $\sigma_{\mathrm{yy}}$ ), are not expected to differ by a random phase between 0 and π . Instead they likely would have opposite phases or equivalently a factor of $e^{i\pi }=-1$ , a π-phase shift. This discrete distribution of SdH phase is supported by the experimental observation in $\rho_{\left(110\right)}$ . The opposite phase scenario is further supported by the harmonic series 40/80/120 T given that all have a π-phase shift, instead of phase shifts of π/2π/3π (*SI Appendix*, Fig. S3).

Here, we explain this π  -phase shift from a semiclassical perspective. We first consider the universal features of open orbits in the superposed lattices. The repetitive application of **Q** in Fig. 1*E* induces both the closed “triangle” orbit and a generic open orbit “…-*v-v-v*-…”. Similar open orbits were constructed from hole ellipsoids and posited to account for the resistivity anisotropy between $\rho_{\mathrm{xx}}$   and $\rho_{\mathrm{yy}}$   (14). Here, with the open orbit parallel to **Q** along the *x*-axis (Fig. 2*A*) and the carriers’ velocity orthogonal to the orbital direction, conduction along the open orbit (oo) is independent of *H* and dominates $\sigma_{\mathrm{yy}}$   as $\sigma_{\mathrm{yy}}∼\sigma_{yy,oo}=\text{const}$   . Other closed orbits (co) only make minor contributions to $\sigma_{\mathrm{yy}}$   that diminish at high field as $\sigma_{\mathrm{yy}}=\sigma_{yy,oo}+\sigma_{yy,co}=\text{const}.+AH^{-2}$   . Therefore, $\rho_{\mathrm{yy}}\left(H\right)$   only depends weakly on *H* and eventually saturates (Fig. 2*C*). By contrast, the open orbit has no effect on $\sigma_{\mathrm{xx}}$   ( $\rho_{\mathrm{xx}}$   ). Magnetoresistivity from closed orbits alone generically evolves with a strong field dependence as $\rho_{\mathrm{xx}}∼H^{2}$   in the Lifshitz-Azbel-Kaganov framework (14, 30, 31); the exponent two is expected in the high field limit. Here our $\rho_{\mathrm{xx}}∼H^{1.3}$ , mainly because sharp corners of DW systems produce a linear form of magnetoresistance (32); our Cr crystals in fields of 14 T are still in the low-field region.

**Fig. 6.**
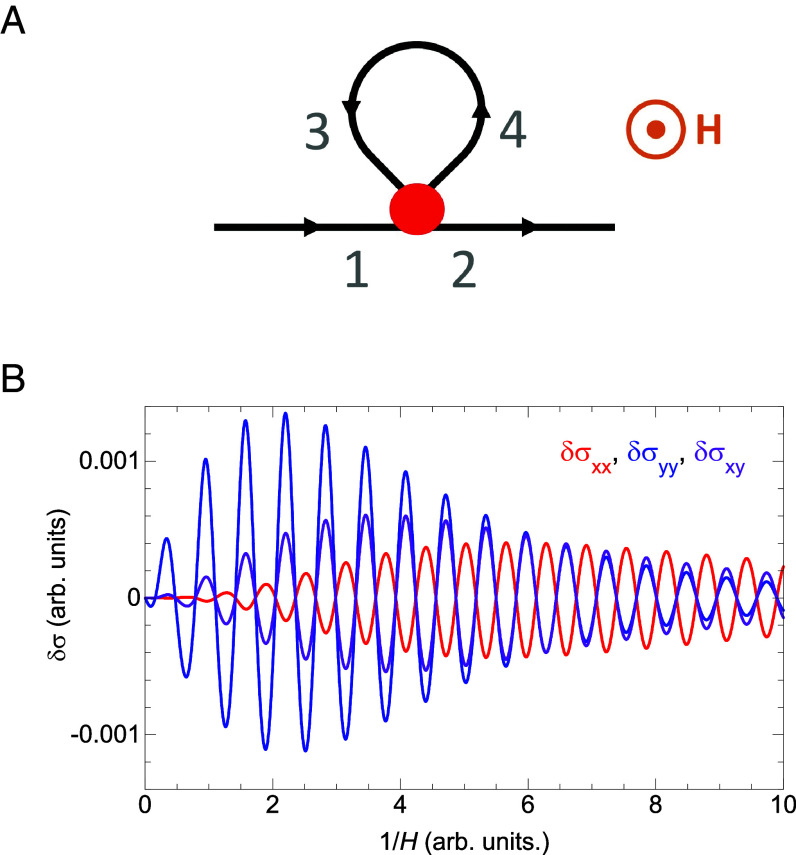
Theoretical model for the π-phase shift. (*A*) Schematics of a minimal model consisting of semiclassical open (1-2, black straight line) and closed (3-4, black loop) orbits, joined together by a quantum mechanical region (red spot). Both open and closed orbits are responsible for transport under field *H* of an incoherent (scattering) nature. The connection region is responsible for coherent SdH oscillations arising from quantum interference, which modulates the transition probability *P* between branches. (*B*) SdH oscillations are simulated using the formulas and parameters given in *SI Appendix*, *Supplementary Note* for all independent conductivity components of σ , using the model structure in panel (*A*). We note $\sigma_{\mathrm{xy}}=-\sigma_{\mathrm{yx}}$ . The opposite phases are clearly demonstrated between $\sigma_{\mathrm{xx}}$ and $\sigma_{\mathrm{yy}}$ . The Hall conductivity $\sigma_{\mathrm{xy}}$ has either a 0 or π phase difference with respect to $\sigma_{\mathrm{xx}}$ and $\sigma_{\mathrm{yy}}$ , but not π/2 as in the integer quantum Hall effect.

A full theoretical treatment of coupled (coherent) quantum orbits and (incoherent) semiclassical galvanomagnetic processes remains challenging because quantum phases along various branches are dependent on the gauge choice of the vector potential **A** (30). Here we construct a composite structure, featuring an open orbit, a closed orbit, both semiclassical (black lines in Fig. 6*A*), and their connection region which incorporates a quantum mechanically coherent network of small orbits or segments (red solid circle in Fig. 6*A*). The small orbits or segments arise from remnants of Fermi surfaces gapped and translated by different harmonics of the SDW and the lattice unit vectors. Such a quantum coherent connection region is equivalent to an effective magnetic breakdown junction (29), with its tunneling probability *P* (see below) oscillates with $H^{-1}$ due to either Landau quantization or quantum interference (27) in the coherent region (30). The salient point is that, for $\rho_{\mathrm{xx}}$ the galvanomagnetic response is dominated by the closed loop, while for $\rho_{\mathrm{yy}}$ the galvanomagnetic response is dominated by the open orbit. The carriers of the closed loop are the leading component of conduction in $\rho_{\mathrm{xx}}$ , but only have a perturbative influence on $\rho_{\mathrm{yy}}$ through the connection region to the (nearly) free carriers along the open orbit. This difference in the identity of the dominant conduction carriers can explain opposite phases in the SdH oscillations.

Carriers along the four branches in Fig. 6*A*, flowing in and out of the quantum region, satisfy the microbalance condition. Because of the closed orbit, currents on branches 3 and 4 are identical under steady-state conditions, and we can denote a probability *P* between 0 to 1 for carriers tunneling between branches 1 and 4, and simultaneously between branches 3 and 2. In the limit of totally decoupled closed and open orbits (*P* = 0), $\sigma_{\mathrm{xx}}$ and $\sigma_{\mathrm{yy}}$ would have the largest difference. As the two channels are joint together and *P* increases, carriers start to mix, and the conductivities become closer in value; $\delta \sigma_{\mathrm{xx}}<0$ and $\delta \sigma_{\mathrm{yy}}<0$ for δP<0 . In other words, by having a finite probability of tunneling, charge carriers on the closed orbit deviate more from the strong-field limit (localization), and carriers on the open orbit become less free. Because of the coherent quantum effect within the connection region, *P* oscillates in addition to monotonically increasing with increasing field. Hence the SdH signatures in $\sigma_{\mathrm{xx}}$ and $\sigma_{\mathrm{yy}}$ have opposite phases. Numerically simulated SdH oscillations are presented in Fig. 6*B* for all independent galvanomagnetic channels. More details of the theoretical framework for solving the coupled orbits problem and of its application to the present model are provided in *SI Appendix*, *Supplementary Note*. We note that in this model SdH oscillations in the Hall conductivity have either a 0 or π phase shift to their counterparts in the magnetoresistance channels, instead of the π /2 difference in the integer quantum Hall effect.

The SdH oscillations of 36 T and 40/80/120 T frequencies are largely different in their galvanomagnetic behavior, for aspects such as harmonics, the field range, and amplitude anisotropy between $\rho_{\mathrm{xx}}$ and $\rho_{\mathrm{yy}}$ . Yet they all demonstrate an opposite phase between two conduction channels. The insensitivity to details can be explained by the generic mechanism described above. Their differences can be attributed to microscopic differences in the semiclassical orbits coupled through different quantum regions (*SI Appendix*, *Supplementary Note*). For example, for SdH oscillations in the Hall conductivity, the fact of carriers being mainly of electron or hole type can shift the phase by 0 or π.

Conventional SdH oscillations reflect the Landau levels’ density of states (26) and those of a linearly dispersive (Dirac) band structure would incorporate an extra π-phase due to the Berry phase (33). In both scenarios, with the current flowing perpendicular to the field, $\sigma_{\mathrm{xx}}$ and $\sigma_{\mathrm{yy}}$ are expected to have identical phases in the SdH oscillations. Importantly, our observed low-frequency SdH oscillations in Cr do not follow these simple scenarios. Instead, the opposite phases between $\rho_{\mathrm{xx}}$ ( $\sigma_{\mathrm{xx}}$ ) and $\rho_{\mathrm{yy}}$ ( $\sigma_{\mathrm{yy}}$ ), contrasted by their congruence in the conventional 1,258 T SdH orbit, reflect an intrinsically anisotropic mechanism of quantum transport. It should be of interest to researchers in the field of topological materials where the phase of SdH oscillations plays a crucial role. In Cr, the two incommensurately superposed lattices naturally create the quantum interference effect, which could be crafted in related quantum and topological materials of varying length scales, from band structure engineering at an interface to multilayers which mediate RKKY interactions and further to photonic crystals.

## Materials and Methods

### Single Q-State in Single-Crystal Cr.

Specimens of single-crystal Cr were procured from two different sources, a wafer of ~2 mm thick and 10 mm diameter with a surface normal of (1, 0, 0) from Alfa Aesar (99.996+%, #13547) (34), and a ~20-mm-long boule of ~5 mm diameter from Atomergic Chemetals Inc. Using single crystals from two sources allows us to avoid potential systematic issues in growth such as trace elements, variable growth parameters, and environment, which could affect the magnetic state. For the Alfa Aesar specimen, the cubic axes within the surface plane were determined by in-lab X-ray diffraction. After the alignment, slices with cubic axes along all surface normals were cut off from the wafer by a diamond saw. All sawed-off surfaces were polished using 50 nm alumina suspension (MicroPolish, Buehler Ltd.), and the pieces were annealed in vacuum inside sealed quartz tubes at 1,000 °C for 32 h using a box furnace. After annealing, the pieces were etched to remove surface oxidation using either a Cr etchant (Type 1020, Transene Co. Inc.) (34), or warm 10% HCl solution. The final piece for the electrical transport study had a length of ~5 mm, with a rectangular cross-section of 1.84 × 0.42 mm^2^ (*SI Appendix*, Fig. S1*A*). Two smaller plates of Cr were prepared for dHvA measurements, with sizes of 1.82 × 1.73 × 0.40 mm^3^ and 1.74 × 1.50 × 0.20 mm^3^, and weight of 10.2 mg and 4.6 mg, respectively. The presence of the SDW/CDW state was confirmed previously by X-ray diffraction studies of other parts of the same wafer (12, 34, 35) and the transport signature at *T*_N_ (Fig. 2*B*) (8). The residual resistivity ratio with I‖Q , ρ (*T* = 350 K)/ρ (*T* = 2 K) ~ 66. The rod-shaped Cr single crystal from Atomergic Chemetals was indexed by a lab Laue diffractometer. Several slices with a rectangular cross-section were cut off from the rod by wire-saw, with the (1, 1, 0) direction along the long direction for the current path, while the side normals are (0, 0, 1) and (1, –1, 0), respectively (*SI Appendix*, Fig. S1*B*). One piece was similarly polished and annealed in vacuum in a quartz tube at 1,000 °C for 48 h, and naturally cooled down in the furnace. Surface oxidation was removed by a warm bath of 10% HCl. The final piece measured ~7.5 × 2.6 × 0.82 mm^3^ (*SI Appendix*, Fig. S1*B*).

Both SdH and dHvA measurements were carried out using a horizontal rotator probe inside a 14 T Physical Property Measurement System (PPMS DynaCool-14, Quantum Design, Inc.). The single Q-state is induced by a slow, controlled field-cooling (0.4 to 0.7 K/min) from 350 K down, using a field of 14 T to align the **Q** vector parallel to **H**. Unlike the cooling rate of the previous single-Q procedure in ref. 15 (~40 min from 340 K to 77 K), our cooling rate is much slower to ensure a stress-free condition of the DW state. While there is a small difference in the procedure between refs. 14 and 15 as whether to reduce the field to zero at ~150 K above the spin-flip transition temperature at ~123 K (36), or at the base temperature ~4 K, we find both procedures work. The single Q-state is reflected by measured SdH/dHvA frequencies. Results from both field-cooling procedures are consistent (*SI Appendix*, Table S1).

### Shubnikov–de Haas Quantum Oscillations.

Each of the two galvanomagnetic samples is anchored to the surface of an 8-pin DIP connector using GE varnish (*SI Appendix*, Fig. S1). Unlike Mg samples in previous studies (30) in which the metallic single crystals are highly susceptible to the cooling and stress relieving condition, Cr is a refractory metal with very high Young’s modulus and shear moduli. The polymer-based surface of the DIP connector does not exert noticeable stress on our single crystals. The sample and DIP connector are mounted onto a PPMS rotator sample carrier, which has been modified with differently positioned DIP sockets to accommodate several in-plane angles of field cooling (such as 0°, 90°, and 45° for Q‖I , Q⊥I , and **I** || (1,1,0) conditions). Electrical leads (Au wire of 25 µm diameter) were attached to the transport samples by silver epoxy (EPO-TEK H20E-PFC, Epoxy Technology) in a six-lead Hall/MR configuration (*SI Appendix*, Fig. S1).

Both magnetoresistance and Hall resistance were measured using an AC resistance bridge and a preamplifier (LS372 and 3708, Lake Shore Cryotronics, Inc.) for the sample inside the PPMS from *T* = 1.65 to 350 K. An electric current of 10 mA was typically used in order to collect low-noise SdH signals. Each matrix element of ρ   was measured under fixed temperature as a function of field, at 40 Oe spacing with 3 to 5 min of averaging time at each field. Given the well-positioned leads placement (*SI Appendix*, Fig. S1) and Hall resistivities being much weaker than MR, the antisymmetric component in our measured MR is less than 1% of the total, so no symmetrization is performed; for Hall resistivities, the data plotted in Fig. 2*D* were antisymmetrized. SdH oscillations were extracted as ${\left(\Delta \rho_{\mathrm{xx}}/\rho_{\mathrm{xx}}\right)}_{SdH}=\left[\rho_{\mathrm{xx}}\left(H\right)-\rho_{\mathrm{xx}}^{\mathrm{BG}}\left(H\right)\right]/\rho_{\mathrm{xx}}\left(H\right)$   for MR and $\Delta \rho_{\mathrm{xy}}=\rho_{\mathrm{xy}}\left(H\right)-\rho_{\mathrm{xy}}^{\mathrm{BG}}\left(H\right)$   for Hall resistivity, where $\rho^{\mathrm{BG}}\left(H\right)$   are smooth functions. Measured SdH oscillations from all matrix elements of ρ   are plotted vs. *H* or 1/*H* in Figs. 3 and 4, together with the Fourier-transformed spectra.

### de Haas–van Alphen Quantum Oscillations.

Magnetization-based dHvA quantum oscillation measurements were carried out using the torque magnetometry technique (*SI Appendix*, Figs. S1 *C* and *D*) based on the Torque Magnetometer option of the PPMS (Tq-Mag, PPMS DynaCool-14, Quantum Design, Inc.). The torque from magnetic anisotropy was detected by piezoresistive elements on the cantilever of Tq-Mag, arranged in a Wheatstone bridge configuration. The change of piezoresistivity is measured by our own AC resistance bridge (LS372 and 3708, Lake Shore Cryotronics, Inc.). In a torque-based dHvA measurement, the continuous cooling to 1.65 K under the 14 T field can be monitored directly and the preservation of the single Q-state is verified across the spin-flip transition; for electrical transport measurements, there is no resistivity signature of the spin-flip transition (8). The measured dHvA oscillations, expressed in the unbalanced resistance of the Wheatstone bridge, were extracted after background subtraction and Fourier transformed to the frequency domain. Two pieces of Cr crystals (10.2 mg and 4.6 mg) in the single Q-state were measured to verify the sample and data consistency.

### Fast Fourier Transform.

FFT analysis was applied to both SdH and dHvA oscillations. For dHvA, as the oscillation amplitudes have a strong field dependence, we used a Tukey window function to clean up the FFT spectrum, which is defined as:

$$\begin{array}{c} w\left(x\right)=\left\{\begin{array}{cc} \frac{1}{2}\left\{1+\text{cos}\left[2\pi \left(\frac{x}{r}-\frac{1}{2}\right)\right]\right\}, & 0\leq x<\frac{r}{2}\\ 1, & \frac{r}{2}\leq x\leq 1-\frac{r}{2}\\ \frac{1}{2}\left\{1+\text{cos}\left[2\pi \left(\frac{x-1}{r}+\frac{1}{2}\right)\right]\right\}, & 1-\frac{r}{2}<x\leq 1 \end{array}.\right. \end{array}$$

In our analysis of dHvA spectra, we chose *r* = 0.1. The Tukey functional form thus affects 5% of the data on the high-field end, as oscillations at the low-field end already decay to the noise floor. This minimally affected range of the window function preserves the relative intensities of different oscillation frequencies, while removing interstitial spectral weight and potential resonance types of spectral anomalies. For SdH oscillations, as the signals have only weak field dependence, a straightforward FFT can be applied.

### Phase Extraction of SdH Oscillations.

For $\rho_{\mathrm{xx}}$   , $\rho_{\mathrm{yy}}$   , and $\rho_{\left(110\right)}$   at 1.65 K, the raw data of ${\left(\Delta \rho /\rho \right)}_{SdH}$   were fit to a functional form of ${\left(\Delta \rho /\rho \right)}_{SdH}=$  $\sum_{F}A_{F}cos\left(2\pi F/H+\psi_{F}\right)exp\left(-K_{F}/H\right)$   , where the oscillation frequencies *F* are fixed from FFT-analyzed spectra. The summation runs through the six strongest oscillation frequencies *F*, which include 36, 40, 80, 120, 1258 T, and either 467 T (for $\rho_{\mathrm{yy}}$ ) or 899 T (for $\rho_{\mathrm{xx}}$ and $\rho_{\left(110\right)}$ ). $K_{F}$ is the decay parameter to capture the exponential field-dependence, similar to the effect of the Dingle temperature.

## Supplementary Material

Appendix 01 (PDF)

## Data Availability

All study data are included in the article and/or *SI Appendix*.
