# Supplementary material for: Quantum interference in superposed lattices
Source: Proc Natl Acad Sci U S A. 2024 Feb 5;121(7):e2315787121. doi: 10.1073/pnas.2315787121 (PMC10873631; doi:10.1073/pnas.2315787121)
Supplement: Supplementary file 1 — Appendix 01 (PDF) [file pnas.2315787121.sapp.pdf]

Supplementary materials for:

Quantum interference in superposed lattices

Yejun Feng[1,*], Yishu Wang[2,3], T. F. Rosenbaum[4], P. B. Littlewood[5,6,7], Hua Chen[8]

[1]Okinawa Institute of Science and Technology Graduate University, Onna, Okinawa 904-0495, Japan
[2]Department of Materials Science and Engineering, University of Tennessee, Knoxville, Tennessee 37996, USA
[3]Department of Physics and Astronomy, University of Tennessee, Knoxville, Tennessee 37996, USA
[4]Division of Physics, Mathematics, and Astronomy, California Institute of Technology, Pasadena, California 91125, USA
[5]The James Franck Institute, The University of Chicago, Illinois 60637, USA
[6]Department of Physics, The University of Chicago, Illinois 60637, USA
[7]School of Physics and Astronomy, University of St Andrews, St Andrews KY16 9SS, United Kingdom
[8]Department of Physics, Colorado State University, Fort Collins, Colorado 80523, USA
*Corresponding author. Email: yejun@oist.jp

This file includes:

Figures S1-S5
Tables S1-S2
Supplementary Note

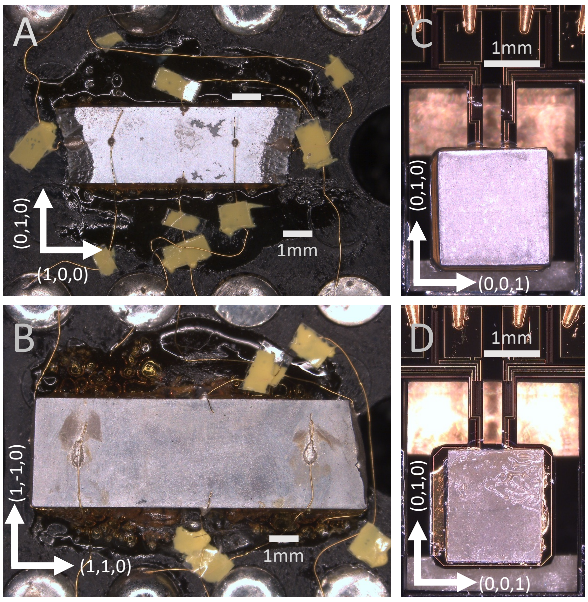

Fig. S1. Pictures of single crystal Cr samples used in (**A-B**) SdH and (**C-D**) dHvA measurements. The two SdH samples, mounted on top of 8-pin DIP connectors, are from (A) Alfa Aesar, and (B) Atomergic Chemetals, respectively (Methods). The two dHvA samples are from Alfa Aesar, and weigh (C) 10.2 and (D) 4.6 mg, respectively. The crystalline orientations are marked alongside the individual sample.

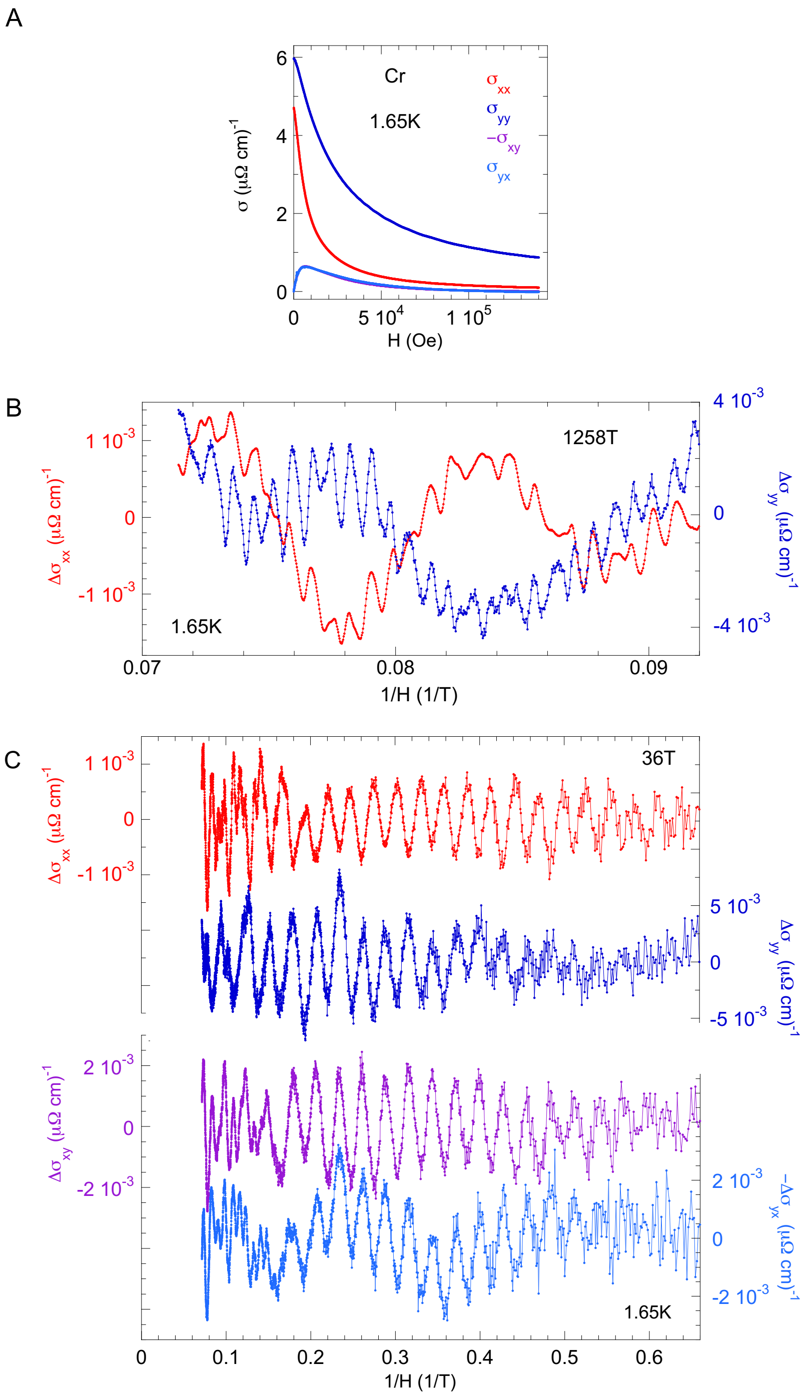

Fig. S2. (A) Electrical conductivity σ calculated from inversion of the resistivity matrix ρ in Fig. 2. (B) 1258 T SdH oscillations in both σ_{xx} and σ_{yy} have the same phase. (C) 36 T SdH oscillations in σ_{xx}, σ_{yy}, σ_{xy}, and $-\sigma_{yx}$, demonstrating the π-phase difference between σ_{xx} and σ_{yy}. The direct verification of the π-phase difference in σ excludes a trivial explanation of matrix inversion from σ to ρ.

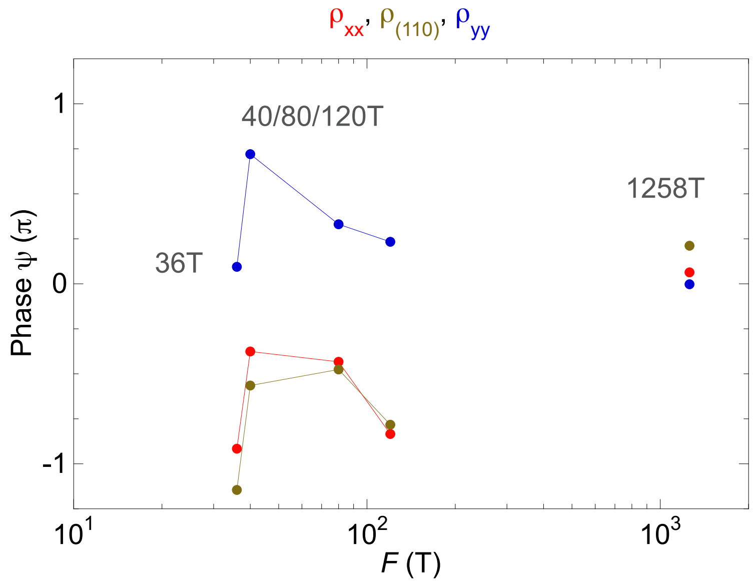

Fig. S3. Extracted SdH phases ψ_F of 36/40/80/120 T in comparison to those of 1258 T (Methods) are plotted for all three magnetoresistivities ρ_{xx}, ρ_{yy}, and $\rho_{(110)}$. The π-phase shift is clear for the low-frequency modes.

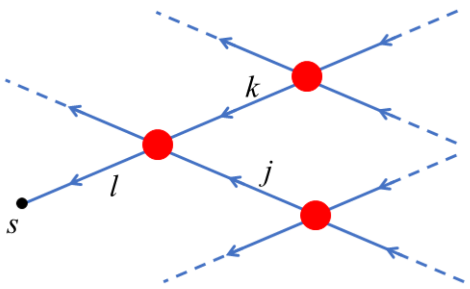

Fig. S4. Schematics of coupled orbits. Each line represents an orbit segment. Red dots stand for magnetic breakdown junctions. s represents the present position of the electron.

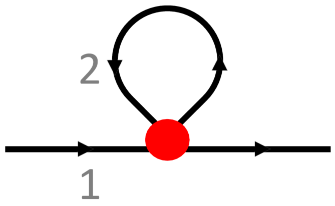

Figure S5. Schematics of a closed and an open orbit coupled through an MB junction.

Table S1. dHvA and SdH frequencies of single-Q Cr under the configuration of H || (0,0,1) and Q || (1,0,0). The dHvA frequencies reported in the literature were extracted from figures in Refs. [15, 23]. For dHvA, only frequencies with intensities higher than 1% of the strongest peak at 899 T are reported here.

Extracted dHvA frequencies from the literature (Tesla)		Measured dHvA frequencies of this work (Tesla)	Measured SdH frequencies from ρ_{xx} (Tesla)	Measured SdH frequencies from $\rho_{(110)}$ (Tesla)
Ref. [15]	Ref. [23]			
32	28	26		
		32	36	36
44	41	44	40	40
		95		
158		158	165	165
		198		
427		428	426	426
		440		
564		547		
		585		
856		862		
903		899	899	898
1229				
1262		1256	1258	1258
1340		1325		
1376		1369	1367	1368
1663		1641		
1768		1755	1752	
1808		1789	1792	1793
2039		2009		
		2186		
2273		2268	2250	2266
		2342	2352	
2392				
		2511	2512	
		2727		
2835				
		2919		
		3278		
3494		3447		
		3529	3540	

Table S2. Parameter values used for getting the numerical results in Fig. 6 of the main text.

ℓ_0^2	0.1
P_0	0.6
P_1	0.1
l_{mfp}	1 (length unit)
s_{B1}	0.2
s_{B2}	1.0
ϕ_0	$-\pi/2$

Supplementary Note: Semiclassical theory for the π-phase shift

The semiclassical Boltzmann equation under the relaxation time approximation has the following form in the steady state:

$$\frac{\partial f}{\partial \mathbf{r}} \cdot \dot{\mathbf{r}} + \frac{\partial f}{\partial \mathbf{k}} \cdot \dot{\mathbf{k}} + \frac{f - f_0}{\tau} = 0, \tag{S1}$$

where τ is the relaxation time and f_0 is the equilibrium Fermi-Dirac distribution function. Following Ref. [31], in the presence of both electric field $\mathbf{E}$ and magnetic field $\mathbf{B}$ the Boltzmann equation can be written as

$$e\tau \frac{\partial f_0}{\partial \epsilon} \mathbf{v} \cdot \mathbf{E} - \frac{\partial g}{\partial s} \frac{v_\perp \tau}{\ell^2} = g, \tag{S2}$$

where $g \equiv f - f_0$, $\ell = \sqrt{\frac{\hbar}{eB}}$ is the magnetic length, $v_\perp$ is the magnitude of velocity in the plane perpendicular to $\mathbf{B}$, and s parameterizes the k-space orbit. Eq. S2 has the following solution:

$$g = \int_{-\infty}^{s} ds' \frac{e\ell^2}{v_\perp} \frac{\partial f_0}{\partial \epsilon} \mathbf{v} \cdot \mathbf{E} \exp\left(-\int_{s'}^{s} \frac{\ell^2}{v_\perp \tau} ds''\right), \tag{S3}$$

using which one can calculate the electric current and hence the conductivity

$$\mathbf{j} = -\sum_n \int \frac{ds\, d\epsilon\, dk_z}{(2\pi)^3} \frac{e}{\hbar v_\perp} \mathbf{v} g. \tag{S4}$$

We next discuss how to generalize Eq. S3 to the case of many orbits coupled through magnetic breakdown (MB) junctions [29]. Each MB junction connects two incoming and two outgoing k-space paths so that each semiclassical particle tunneling through the MB junction has a nonzero possibility of choosing either of the two outgoing paths. Therefore Eq. S3 must be generalized to a summation over all possible trajectories of a given particle weighted by their respective probabilities:

$$g = \sum_p P_p \int_{-\infty_p}^{s_p} ds_p' \frac{e\ell^2}{v_\perp} \frac{\partial f_0}{\partial \epsilon} \mathbf{v} \cdot \mathbf{E} \exp\left(-\int_{s_p'}^{s_p} \frac{\ell^2}{v_\perp \tau} ds_p''\right), \tag{S5}$$

where p stands for path and P_p is the probability of taking a given path. To perform such a path summation, we label all directed segments of the couple orbits by l, j, k etc. as illustrated in Fig. S4. Due to the definite directions of the segments, the starting point of a given segment l can be uniquely denoted by s_l, which coincides with the starting point of another segment going out of the same MB junction as l. To calculate $g(s)$ with the s indicated in the figure, note that the last part of all paths coincide:

$$g(s) = \int_{s_l}^{s} ds' F_1(s') e^{-\int_{s'}^{s} F_2(s'')ds''} + e^{-\int_{s_l}^{s} F_2(s'')ds''} I_l, \tag{S6}$$

where

$$F_1 \equiv \frac{e\ell^2}{v_\perp} \frac{\partial f_0}{\partial \epsilon} \mathbf{v} \cdot \mathbf{E}, \tag{S7}$$

$$F_2 \equiv \frac{\ell^2}{v_\perp \tau},$$

$$I_l \equiv \sum_p P_p \int_{-\infty_p}^{s_l} ds_p' F_1(s_p') e^{-\int_{s_p'}^{s_l} F_2(s_p'')ds_p''}.$$

The key is therefore to calculate I_l. However, note that there are only two segments (k, j in Fig. S4) that go into the MB junction from which l leaves. Assuming I_l is well-defined for all l, we have

$$I_l = P_{l\leftarrow k}\left[\int_{s_l-s_{Bk}}^{s_l} ds' F_1(s') e^{-\int_{s'}^{s_l} F_2(s'')ds''} + e^{-\int_{s_l-s_{Bk}}^{s_l} F_2(s')ds'} I_k\right] \quad \text{(S8)}$$
$$+P_{l\leftarrow j}\left[\int_{s_l-s_{Bj}}^{s_l} ds' F_1(s') e^{-\int_{s'}^{s_l} F_2(s'')ds''} + e^{-\int_{s_l-s_{Bj}}^{s_l} F_2(s')ds'} I_j\right],$$

where $P_{l\leftarrow k}$ is the probability that the electron in segment l comes from segment k at the MB junction, s_{Bk} is the length of segment k. The above equation can be written as a matrix equation

$$\mathbf{I} = \mathrm{M}\cdot\mathbf{V} + \mathrm{M}\cdot\mathrm{C}\cdot\mathbf{I}, \quad \text{(S9)}$$

where

$$\mathrm{M}_{lk} = P_{l\leftarrow k} \quad \text{(S10)}$$
$$V_l = \int_{s_l^{\mathrm{end}}-s_{Bl}}^{s_l^{\mathrm{end}}} ds' F_1(s') e^{-\int_{s'}^{s_l^{\mathrm{end}}} F_2(s'')ds''}$$
$$\mathrm{C}_{lk} = \delta_{lk} e^{-\int_{s_l^{\mathrm{end}}-s_{Bl}}^{s_l^{\mathrm{end}}} F_2(s')ds'}.$$

The s_l^{end} in the above equations mean the s-coordinate of the end point of segment l. Eq. S9 is reminiscent of the Dyson equation and can be immediately solved

$$\mathbf{I} = (\mathbb{I} - \mathrm{MC})^{-1}\mathrm{M}\mathbf{V} \quad \text{(S11)}$$

We now use the above formalism to solve the problem of two coupled orbits illustrated in Fig. S5. We label the open orbit as 1 and the closed one as 2, both being electron-like, and with the magnetic field pointing out of the paper. At the MB junction we have

$$\mathrm{M} = \begin{pmatrix} Q & P \\ P & Q \end{pmatrix}, \quad (12)$$

where $Q \equiv 1 - P$. $P = 1$ means complete breakdown and $P = 0$ means no breakdown. Eq. S11 then gives

$$\begin{pmatrix} I_1 \\ I_2 \end{pmatrix} = \frac{1}{(1-QC_1)(1-QC_2)-P^2C_1C_2}\begin{pmatrix} (1-QC_2)(QV_1+PV_2)+PC_2(PV_1+QV_2) \\ PC_1(QV_1+PV_2)+(1-QC_1)(PV_1+QV_2) \end{pmatrix}, \quad (13)$$

where $C_{1,2}$ are the diagonal elements of the C matrix. One can readily check that the above solution reduces to the known results based on Eq. S3 for the special cases of $P = 0$ (no MB) and $P = 1$ (complete MB), for which we give the results of $I_{1,2}$ to be used below:

$$(I_1, I_2) = \begin{cases} \left(\frac{V_1}{1-C_1}, \frac{V_2}{1-C_2}\right) & P = 0 \\ \left(\frac{V_2+C_2V_1}{1-C_1C_2}, \frac{V_1+C_1V_2}{1-C_1C_2}\right) & P = 1 \end{cases}. \quad (14)$$

When the MB junction is replaced by an effective one incorporating a small network of coherent paths, P generally acquires an oscillatory part versus H^{-1} [30],

which can be due to either Landau quantization or quantum interference. Below we show that for the present toy model, an oscillation in P leads to oscillations of σ_{xx} and σ_{yy} whose phases are different by π.

We first give a qualitative discussion by comparing the cases of $P = 0$ and $P = 1$ for σ_{xx} and σ_{yy} separately.

(1) σ_{yy} ($\mathbf{E} \perp \mathbf{Q}$)

We consider the high-field regime and assume the mean-free path $l_{\mathrm{mfp}} = v_\perp \tau$ to be constant for simplicity. Then

$$C_{1,2} \approx 1 - 2\pi(\omega_{c1,2}\tau)^{-1} \tag{15}$$
$$V_{1,2} \approx \int_{0_{1,2}}^{0_{1,2}+s_{B1,2}} ds' F_1(s') \left[1 - \frac{(s_{B1,2}+0_{1,2}-s')\ell^2}{l_{\mathrm{mfp}}}\right],$$

where $\omega_{c1,2} \equiv \frac{2\pi v_\perp}{\ell^2 s_{B1,2}}$. For V_1 it is sufficient to keep the lowest order term since $\mathbf{v} \cdot \mathbf{E} \neq 0$ on the open orbit and its integral over the open orbit does not vanish. For V_2 the lowest order term vanishes [31].

For $P = 0$, the two orbits are decoupled, and for σ_{yy} up to $O(H^{-1})$ we only need to consider the contribution due to the open orbit. Using Eq. S14 one can get

$$g_1(s) \approx g_1^{(0)}(s) = \frac{\omega_{c1}\tau}{2\pi} V_1 = \frac{e\tau}{s_{B1}} \oint_1 ds' \frac{\partial f_0}{\partial \epsilon} \mathbf{v} \cdot \mathbf{E}, \tag{16}$$

where the subscript 1 means the argument s of $g_1(s)$ belongs to the open orbit, $g_1^{(0)}(s)$ means the contribution to $g_1(s)$ that is $O(H^0)$.

On the other hand, when $P = 1$, Eq. S14 suggests that

$$I_1 \approx I_2 \approx \frac{V_1+V_2}{1-C_1C_2}. \tag{17}$$

As a result,

$$g_1^{(0)}(s) = g_2^{(0)}(s) = \frac{(2\pi)^{-1}}{(\omega_{c1}\tau)^{-1}+(\omega_{c2}\tau)^{-1}} V_1 = \frac{e\tau}{s_{B1}+s_{B2}} \oint_1 ds' \frac{\partial f_0}{\partial \epsilon} \mathbf{v} \cdot \mathbf{E}. \tag{18}$$

Therefore,

$$\frac{\sigma_{yy}(P=1)-\sigma_{yy}(P=0)}{\sigma_{yy}(P=0)} = -\frac{s_{B2}}{s_{B1}+s_{B2}}, \tag{S19}$$

which is always negative. Therefore for the open orbit, a finite MB probability always suppresses the conductivity perpendicular to the open orbit because the closed orbit simply increases the effective dissipation when electrons or holes from the open orbit tunnel into it, but does not contribute to current generation in the high-field limit since the carriers excited by the electric field on the closed orbit cancel out.

(2) σ_{xx} ($\mathbf{E} \parallel \mathbf{Q}$)

In this case $V_1 = 0$ since $\mathbf{v} \cdot \mathbf{E} = 0$. Therefore, when $P = 0$ only the closed orbit contributes to $\sigma_{xx} \sim H^{-2}$. When $P = 1$, the open orbit contributes to σ_{xx} only through $I_2 = \frac{C_1 V_2}{1-C_1 C_2}$. As a result, the net change of $g_2(s)$ is

$$\delta g_2(s) = e^{-\int_{0_2}^{s} F_2(s')ds'} \left(-\frac{V_2}{1-C_2} + \frac{C_1 V_2}{1-C_1 C_2}\right). \tag{20}$$

However, V_2 in general has a finite $O(H^{-1})$ term. Assuming the closed orbit to be circular, we have

$$\begin{aligned} V_2 &= eE\ell^2 \frac{\partial f_0}{\partial \epsilon} \int_{\phi_0}^{\phi_0+2\pi} d\phi \frac{s_{B2}}{2\pi} \cos\phi e^{-\frac{\phi_0+2\pi-\phi}{\omega_{c2}\tau}} \\ &\approx eE\ell^2 \frac{\partial f_0}{\partial \epsilon} \frac{s_{B2}}{\omega_{c2}\tau} \sin\phi_0, \end{aligned} \tag{21}$$

where ϕ_0 is the angular coordinate of the MB junction on orbit 2. Then one can obtain

$$\delta g_2(s) \approx e^{-\frac{\phi_s-\phi_0}{\omega_{c2}\tau}} eEl_{\text{mfp}} \frac{\partial f_0}{\partial \epsilon} (\omega_{c2}\tau)^{-1} \sin\phi_0 \left(-\frac{s_{B1}}{s_{B1}+s_{B2}}\right) \tag{22}$$

To get the contribution from $\delta g_2(s)$ to the current, we use Eq. S4 and for simplicity consider a 2D system:

$$\mathbf{j} = -\int \frac{dsd\epsilon}{(2\pi)^2} \frac{e}{\hbar} \hat{v}(s) g(s,\epsilon). \tag{23}$$

We also consider zero temperature so that $\partial_\epsilon f_0 = -\delta(\epsilon - \epsilon_F)$. To perform the s integral one needs to pay attention to how to convert s defined for the two orbits joined together into angular variable ϕ. The resulting $\delta\sigma_{xx}$ is

$$\begin{aligned} \delta\sigma_{xx} &= \frac{e^2}{h} \frac{1}{E} \int_{\phi_0}^{\phi_0+2\pi} \frac{s_{B2}}{(2\pi)^2} \cos(\phi_s) \delta g_2(\phi_s) d\phi_s \\ &\approx \frac{e^2}{h} \frac{s_{B2} l_{\text{mfp}}}{2\pi} (\omega_{c2}\tau)^{-2} \left(\frac{s_{B1}}{s_{B1}+s_{B2}}\right) \sin^2\phi_0, \end{aligned} \tag{24}$$

which is of the same order $O(H^{-2})$ as $\sigma_{xx}(P = 0)$ and is, more importantly, non-negative. Therefore as long as $\phi_0 \neq 0$, i.e. the open orbit is not exactly bisecting the closed one, a finite tunneling probability P can enhance σ_{xx}.

The reason for the non-negative $\delta\sigma_{xx}$ can be understood in the following way. Different from the open orbit, for which a higher dissipation always suppresses conductivity, the conductivity for the closed orbit increases when $\omega_c\tau$ becomes smaller in the high-field limit. Since the only effect of the open orbit for σ_{xx} is to increase dissipation, in the high-field limit coupling to it can make the conductivity for the closed orbit larger.

We finally consider the Hall conductivities. For simplicity we only discuss σ_{yx} below since it can be shown for the present model that $\sigma_{xy} = -\sigma_{yx}$. In this case V_1 vanishes identically. But since the velocity along y for the open orbit is nonzero, it can contribute to σ_{yx} when $P = 1$. Namely, $g_1(s)$ becomes nonzero when $P = 1$. Using Eq. S21, we obtain:

$$\delta g_1(s) = e^{-\frac{s\ell^2}{l_{\text{mfp}}}} \frac{V_2}{1-C_1 C_2} \tag{25}$$

$$\approx e^{-\frac{s\ell^2}{l_{\mathrm{mfp}}}} \frac{s_{B2}}{s_{B1}+s_{B2}} l_{\mathrm{mfp}}(\omega_{c2}\tau)^{-1}\sin\phi_0.$$

On the other hand, $\delta g_2(s)$ is given by Eq. S22 and is already $O(H^{-1})$, which will lead to an $O(H^{-2})$ contribution to the current after integration over the closed orbit. Therefore we only need to consider the current or $\delta\sigma_{yx}$ due to δg_1:

$$\delta\sigma_{yx} = -\frac{e^2}{h}\frac{s_{B2}}{s_{B1}+s_{B2}} l_{\mathrm{mfp}}(\omega_{c2}\tau)^{-1}\sin\phi_0 \int_0^{s_{B1}} \frac{ds}{2\pi} e^{-\frac{s\ell^2}{l_{\mathrm{mfp}}}} \approx -\frac{e^2}{h}\frac{(s_{B2})^2 s_{B1}}{(2\pi)^2(s_{B1}+s_{B2})}\ell^2\sin\phi_0, \quad (26)$$

which can be shown to be identical to $-\delta\sigma_{xy}$. The sign of $\delta\sigma_{xy}$ or $\delta\sigma_{yx}$ is therefore non-universal as it depends on the sign of $\sin\phi_0$ in addition to the carrier types of the two orbits. In the present model, when $\sin\phi_0 < 0$, $\delta\sigma_{xy}$ and $\delta\sigma_{yy}$ are in phase and are out of phase with $\delta\sigma_{xx}$.

Numerical results in Fig. 6b of the main text were obtained using Eqs. S6 and S13 by considering a 2D system and zero temperature, assuming $P = P_0 + P_1\cos(\ell^2/\ell_0^2)$, with the other parameter values given in Table S2. The conductivities thus have the units of e^2/h and are plotted against $\ell^2/l_{\mathrm{mfp}}^2$.
